# DISPEL: database for ascertaining the best medicinal plants to cure human diseases

**DOI:** 10.1093/database/baad073

**Published:** 2023-10-16

**Authors:** Kavya Singh, Harshit Maurya, Parthasarathi Singh, Pujarani Panda, Amit Kumar Behera, Arshad Jamal, Ganesh Eslavath, Somesh Mohapatra, Harsh Chauhan, Deepak Sharma

**Affiliations:** Computational Biology and Translational Bioinformatics (CBTB) Laboratory, Department of Biosciences and Bioengineering, Indian Institute of Technology Roorkee, Roorkee, Uttarakhand 247667, India; Computational Biology and Translational Bioinformatics (CBTB) Laboratory, Department of Biosciences and Bioengineering, Indian Institute of Technology Roorkee, Roorkee, Uttarakhand 247667, India; Department of Computer Science and Engineering, Indian Institute of Technology Roorkee, Roorkee, Uttarakhand 247667, India; Computational Biology and Translational Bioinformatics (CBTB) Laboratory, Department of Biosciences and Bioengineering, Indian Institute of Technology Roorkee, Roorkee, Uttarakhand 247667, India; Computational Biology and Translational Bioinformatics (CBTB) Laboratory, Department of Biosciences and Bioengineering, Indian Institute of Technology Roorkee, Roorkee, Uttarakhand 247667, India; Computational Biology and Translational Bioinformatics (CBTB) Laboratory, Department of Biosciences and Bioengineering, Indian Institute of Technology Roorkee, Roorkee, Uttarakhand 247667, India; Computational Biology and Translational Bioinformatics (CBTB) Laboratory, Department of Biosciences and Bioengineering, Indian Institute of Technology Roorkee, Roorkee, Uttarakhand 247667, India; Computational Biology and Translational Bioinformatics (CBTB) Laboratory, Department of Biosciences and Bioengineering, Indian Institute of Technology Roorkee, Roorkee, Uttarakhand 247667, India; Computational Biology and Translational Bioinformatics (CBTB) Laboratory, Department of Biosciences and Bioengineering, Indian Institute of Technology Roorkee, Roorkee, Uttarakhand 247667, India; Plant Molecular Biology and Biotechnology Laboratory, Department of Biosciences and Bioengineering, Indian Institute of Technology Roorkee, Roorkee, Uttarakhand 247667, India; Computational Biology and Translational Bioinformatics (CBTB) Laboratory, Department of Biosciences and Bioengineering, Indian Institute of Technology Roorkee, Roorkee, Uttarakhand 247667, India

## Abstract

Medicinal plants are anticipated to be one of the most valuable resources for the remedial usage in the treatment of various ailments. The data on key medicinal plants and their therapeutic efficacy against various ailments are quite scattered and not available on a single platform. Moreover, currently there is no means/mechanism of finding the best medicinal plant(s) from numerous plants known to cure any disease. DISPEL (Diseases Plants Eliminate) is a compendium of medicinal plants available across the world that are used to cure infectious as well as non-infectious diseases in humans. The association of a medicinal plant with a disease it cures is hereby referred to as ‘medicinal plant–disease cured’ linkage. The DISPEL database hosts ∼60 000 ‘medicinal plant–disease cured’ linkages encompassing ∼5500 medicinal plants and ∼1000 diseases. This platform provides interactive and detailed visualization of medicinal plants, diseases and their relations using comprehensible network graph representation. The user has the freedom to search the database by specifying the name of disease(s) as well as the scientific/common name(s) of plant. Each ‘medicinal plant–disease cured’ relation is scored based on the availability of any medicine/product derived from that medicinal plant, information about active compound(s), knowledge regarding the part of plant that is effective and number of distinct articles/books/websites confirming the effectiveness of the medicinal plant. The user can find the best plant(s) that can be used to cure any desired disease(s). The DISPEL database is the first step towards generating the ‘most-effective’ combination of plants to cure a disease since it delineates as well as ranks all the therapeutic medicinal plants for that disease. The combination of best medicinal plants can then be used to conduct clinical trials and thus pave the way for their use in clinics for treatment of diseases.

**Database URL** https://compbio.iitr.ac.in/dispel

## Introduction

Since prehistoric times, therapeutic plants have played a vital role in the treatment of diseases, thus, sustaining population health (1, 2). Plants synthesize various significant phytochemicals that operate as a defence mechanism against pathogens, insects, fungi, bacteria and other threats (3–8). Medicinal plants are therapeutically significant for curing various ailments such as hypertension, diabetes, malaria, flu, diarrhea, cancer, etc. (9–14). Towards this end, plant extracts are employed in raw, unprocessed or modified forms. Active components isolated from therapeutic plants have been utilized to make synthetic medicines, such as quinine and reserpine (15, 16).

The information regarding medicinal plants is scattered amongst several research publications and books. Hence, the usage of medicinal plants for therapeutic applications becomes empirically challenging (17, 18). A comprehensive resource of medicinal plants, their therapeutic use, part of the plant involved and active ingredients would be quite useful for drug discovery as well as clinical applications. The databases that have been developed till date are typically country-specific (for instance, TCMID for Chinese medicinal plant (19), MPDB for Bangladeshi medicinal plants (20)], phytochemical-specific [such as Phytochemica (21), IMPPAT (22), TCM-Mesh (23)]) or disease-specific (for instance, CVDHD (24), NPACT (25)). However, the most challenging task is how to choose the best medicinal plant(s) from the myriad of plants known to play a role in curing any specified disease.

Hence, we have built DISPEL (Diseases Plants Eliminate) database which is a comprehensive resource of medicinal plants available across the globe that are used for the treatment of infectious as well as non-infectious diseases in humans. The database hosts >60 000 ‘medicinal plant**–**disease cured’ linkages encompassing ∼5500 medicinal plants and ∼1000 diseases. The DISPEL database can be utilized in diverse cases and scenarios *viz.* a single-faceted study of a particular plant/disease as well as comparative analysis pertaining to heterogeneous assortment of multiple plants/diseases. The server uses a network graph model to provide dynamic and detailed visualization of medicinal plants, diseases and their relationships. The user has the option to search the database by entering the scientific/common name of the plant(s) or the name of the disease(s). Most significantly, the user can find the most effective plants that can be utilized to treat any desired diseases.

## Methodology

### Data collection, preprocessing and extraction

**Figure 1.**
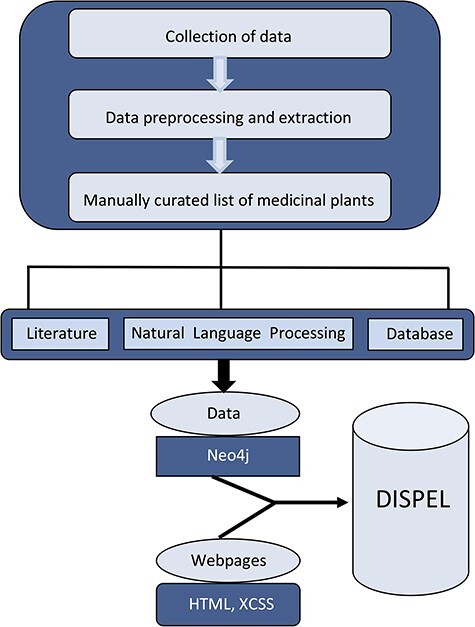
Schematic representation of overall strategy used in the construction of DISPEL database.

**Figure 2.**
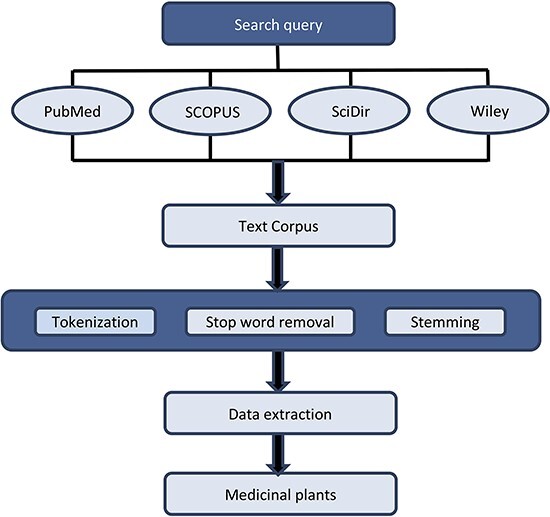
Flow chart of data preprocessing and extraction. Preprocessing involves three main steps: (i) Tokenization, (ii) Stop word removal and (iii) Stemming. For data extraction, Python and Natural Language Toolkit were used.

DISPEL database collates a variety of information on medicinal plants, including their scientific/common name, classification, habitat, country, the part of the plant that is used to treat the ailment, the compound/medicine (if available) and the therapeutic properties (Figure 1). Towards this end, we have compiled information from numerous publications/books/websites and assembled a comprehensive list of more than 5500 medicinal plants (Figure 2). Additional searches for the plant’s scientific/common names were conducted on PubMed (http://www.ncbi.nlm.nih.gov/pubmed) in order to find relevant data on the therapeutic herbs.

### Database architecture and web interface

At the core of the DISPEL database lies the Neo4j software, which is a graph database management system (26) and it has been preferred over other database engines as the data to be stored/retrieved by the application is inherently graphical, i.e. plant nodes, disease nodes and their relationships (Figure 1). Hence, the data entities and relationships get perfectly modeled with Neo4j graph database. Neo4j provides constant time traversals for both depth and breadth due to the efficient representation of nodes and relationships. This ensures efficient scaling for a large number of nodes for the DISPEL database. Cypher Query Language has been employed as the query language for Neo4j database which aids in generating complex queries requesting graphs for multiple plants and diseases. Appropriate cypher query has been used to load the data into the Neo4j server without introducing any redundancies.

A plant entity consists of its name and a number of additional attributes such as its classification, habitat and country. The only component of a disease entity is its name, while a relationship entity consists of the score factor which is calculated based on the plant part (extract) that effectively treats the disease, compound/medicine (if available) and article/book/website references. The DISPEL server interface permits full-text searching with tolerance for typographical errors, for which full-text indices have been created for both plant and disease attributes. The full-text search is a fuzzy string search that uses the Levenshtein algorithm for closeness matching (27). A full-text query attempts to find five plants/diseases with closely matching names.

The backend server has been written in JavaScript using the Express.js library for REST API development (28). JSON strings have been used for bidirectional message transfer between backend and frontend (29). The Node.js application sends cypher queries to the Neo4j server using the Neo4j-driver library and obtains the results as JavaScript objects (30). The results are further processed and sent to the frontend where they are appropriately rendered. Two types of queries, *viz.* graph data queries and search queries can be performed. The graph query fetches graph data from Neo4j server and sends the serialized object to the frontend client. The graph query accepts plant identifiers, disease identifiers and mode (center of interest: plant/disease) to return graph data. The search query fetches the names of plants/diseases and sends a list of search results to the client. The open-source Model-View-ViewModel (MVVM) JavaScript framework called Vue.js was used to create frontend user interfaces (31). The server has the ability to handle multiple requests concurrently.

## Results and discussion

**Figure 3.**
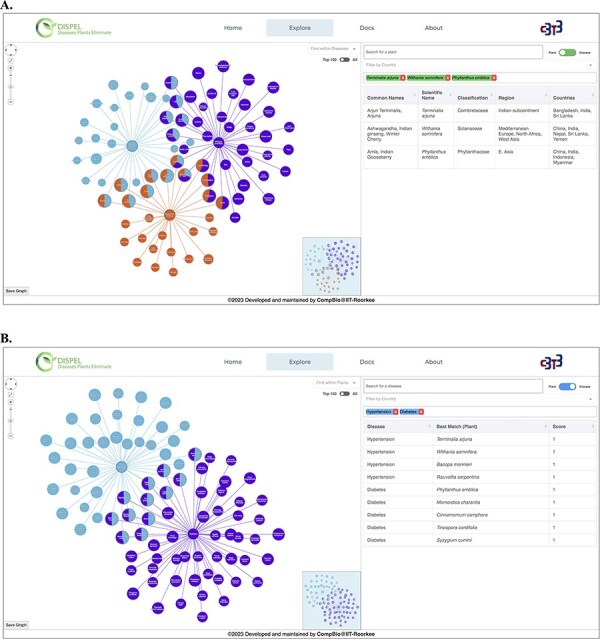
(A) Representative output of ‘Plant Search’ using three medicinal plants (center nodes): *Terminalia arjuna* (Arjuna, light blue), *Withania somnifera* (Ashwagandha, purple) and *Phyllanthus emblica* (Amla, orange). The diseases are shown as leaf/edge nodes. (B) Representative output of ‘Disease Search’ using two diseases (center nodes): hypertension (light blue) and diabetes (purple).

We have built a user-friendly interface DISPEL to access the enormous, untapped data on medicinal plants encompassing ∼60 000 linkages of plants and human maladies that can be cured. The DISPEL database can be utilized in diverse cases *viz.* single-faceted study of a particular plant/disease and comparative analysis of multiple plants/diseases. This platform provides interactive and detailed visualization of medicinal plants, diseases and their relations using comprehensible network graph representation (Figure 3). Each ‘medicinal plant**–**disease cured’ relation is scored based on the availability of any medicine/product based on that medicinal plant, information about active compound(s), knowledge regarding the part of the plant that is effective and number of distinct articles/books/websites confirming the effectiveness of the medicinal plant. A medicinal plant with a score of 1 is regarded as the best plant for treating a certain ailment.

**Figure 4.**
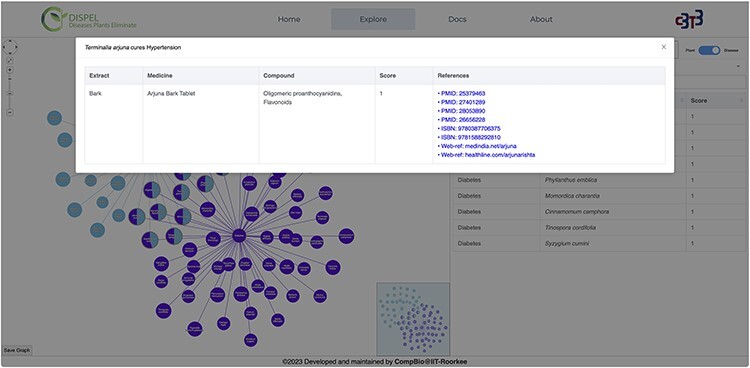
Clicking on the ‘*Terminalia arjuna*-hypertension’ link/edge displays the relevant information about the extract (part of plant), medicine, compound, score and references.

The user has the freedom to search the database by specifying the name of disease(s) as well as the scientific/common name(s) of the plant (Figure 3). While typing, the search engine will automatically provide a list of plants or diseases that fits the query. The resultant graph (Figure 3) depicts relationships between plants and their therapeutic usage (diseases). The graph is interactive and the nodes can be moved around as per the convenience of the user. The search bar present in the graphical network (in left hand side section) can be used to find any desired plants/diseases within the displayed network. User can search one or multiple plants/diseases simultaneously and the nodes searched will become apparent (blink) in the graphical network. Furthermore, a country search feature has also been incorporated, allowing users to search for one or more plants from a specific country. Hovering on the edges displays the score of the relation between the medicinal plant and the disease cured; the radius of the node is proportional to the score. Clicking on the edges will display all the relevant information about that ‘medicinal plant**–**disease cured’ link (Figure 4). Furthermore, hovering over plant nodes shows detailed information about that specific plant. Most importantly, during ‘Disease Search’, the best medicinal plant(s) (with the highest score) for each disease will be displayed in a tabular manner (Figure 3B). The user also has the option to view all the plants known to cure a desired disease. The graph panel also includes handy controls to pan across as well as zoom in and out of the graph.

To exemplify the utility of DISPEL server, we searched for medicinal plants curing hypertension and diabetes (Figure 3B). The best medicinal plants for curing each of the diseases get retrieved/displayed. The best plants for treating hypertension are *Terminalia arjuna* (Arjuna), *Withania somnifera* (Ashwagandha), *Bacopa monnieri* (Brahmi) and *Rauvolfia serpentina* (Indian snakeroot) while those for curing diabetes are *Phyllanthus emblica* (Amla), *Momordica charantia* (Karela), *Cinnamomum camphora* (Kapur), *Tinospora cordifolia* (Giloy) and *Syzygium cumini* (Jamun). Furthermore, plants effective in curing both the diseases simultaneously are also delineated. *Zingiber officinale* (Black Ginger), *Punica granatum* (Pomegranate) and *Terminalia arjuna* (Arjuna) are the best plants for concurrent treatment of diabetes and hypertension (Figure 3B). On the other hand, we can also use the ‘Plant Search’ feature in DISPEL to explore the diseases cured by distinct medicinal plants. For instance, *Phyllanthus emblica, Terminalia arjuna* and *Withania somnifera* can be combined together to cure diabetes as well as snake bite (Figure 3A). Thus, DISPEL database enumerates the ‘most-effective’ combination of plants to cure any desired disease(s). The combination of best medicinal plants can then be used to conduct clinical trials, and thus lead to the development of new, efficacious natural products for the treatment of diseases.

## Conclusion

The user can find the best plant(s) that can be used to cure any desired disease(s). The DISPEL database will be helpful in determining the ‘most-effective’ combination of plants to cure a disease. Subsequently, clinical trials may be conducted to pave the way for the use of medicinal plant therapies in clinics for treatment of diseases. This resource will be beneficial to plant/medical scientists for discovering novel, natural treatment regimens.
